# Hepatic Lactate Dehydrogenase A: An RNA Interference Target for the Treatment of All Known Types of Primary Hyperoxaluria

**DOI:** 10.1016/j.ekir.2021.01.029

**Published:** 2021-02-03

**Authors:** Gema Ariceta, Kelly Barrios, Bob D. Brown, Bernd Hoppe, Ralf Rosskamp, Craig B. Langman

**Affiliations:** 1 Division of Pediatric Nephrology, Hospital Universitari Vall d’Hebron, Universitat Autonoma de Barcelona, Barcelona, Spain; 2 Nefrología Pediátrica, Hospital Infantil, Hospital Universitari Vall d’Hebron, Passeig de la Vall d’Hebron, Barcelona, Spain; 3 Dicerna Pharmaceuticals, Inc., Lexington, Massachusetts, USA; 4 German Hyperoxaluria Center Cologne/Bonn, Bonn, Germany; 5 Feinberg School of Medicine, Northwestern University, Chicago, Illinois, USA; 6 Ann and Robert H. Lurie Children’s Hospital of Chicago, Chicago, Illinois, USA

**Keywords:** lactate dehydrogenase A, nedosiran, primary hyperoxaluria, RNA interference, small interfering RNA

## Abstract

**Introduction:**

Primary hyperoxaluria (PH) is a family of 3 rare genetic disorders of hepatic glyoxylate metabolism that lead to overproduction and increased renal excretion of oxalate resulting in progressive renal damage. *LDHA* inhibition of glyoxylate-to-oxalate conversion by RNA interference (RNAi) has emerged as a potential therapeutic option for all types of PH. *LDHA* is mainly expressed in the liver and muscles.

**Methods:**

Nonclinical data in mice and nonhuman primates show that *LDHA* inhibition by RNAi reduces urinary oxalate excretion and that its effects are liver-specific without an impact on off-target tissues, such as the muscles. To confirm the lack of unintended effects in humans, we analyzed data from the phase I randomized controlled trial of single-dose nedosiran, an RNAi therapy targeting hepatic *LDHA*. We conducted a review of the literature on LDHA deficiency in humans, which we used as a baseline to assess the effect of hepatic *LDHA* inhibition.

**Results:**

Based on a literature review of human LDHA deficiency, we defined the phenotype as mainly muscle-related with no liver manifestations. Healthy volunteers treated with nedosiran experienced no drug-related musculoskeletal adverse events. There were no significant alterations in plasma lactate, pyruvate, or creatine kinase levels in the nedosiran group compared with the placebo group, signaling the uninterrupted interconversion of lactate and pyruvate and normal muscle function.

**Conclusion:**

Phase I clinical data on nedosiran and published nonclinical data together provide substantial evidence that *LDHA* inhibition is a safe therapeutic mechanism for the treatment of all known types of PH.

## Graphical abstract

**Figure:**
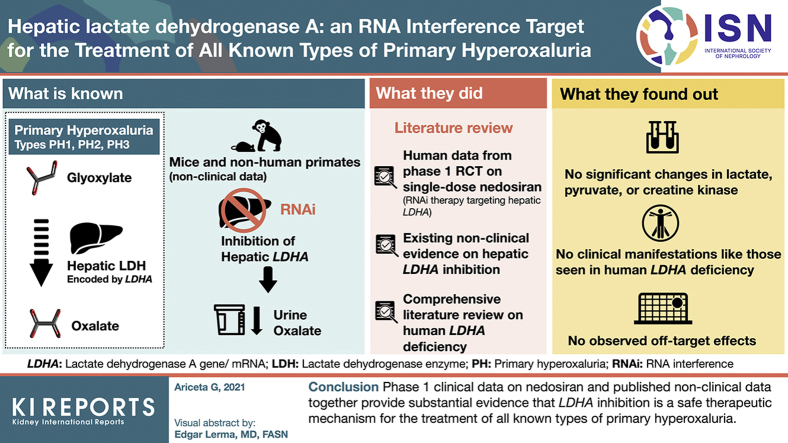

Primary hyperoxaluria (PH) is a family of rare, autosomal recessive genetic disorders of glyoxylate metabolism resulting in overproduction of oxalate in the liver.^1^^,^^2^ Three genetic types of PH (PH1, PH2, and PH3) have been defined, each caused by mutations that result in a deficiency, mislocalization, or loss-of-function of key enzymes (Figure 1) involved in glyoxylate metabolism.^3^, ^4^, ^5^

**Figure 1:**
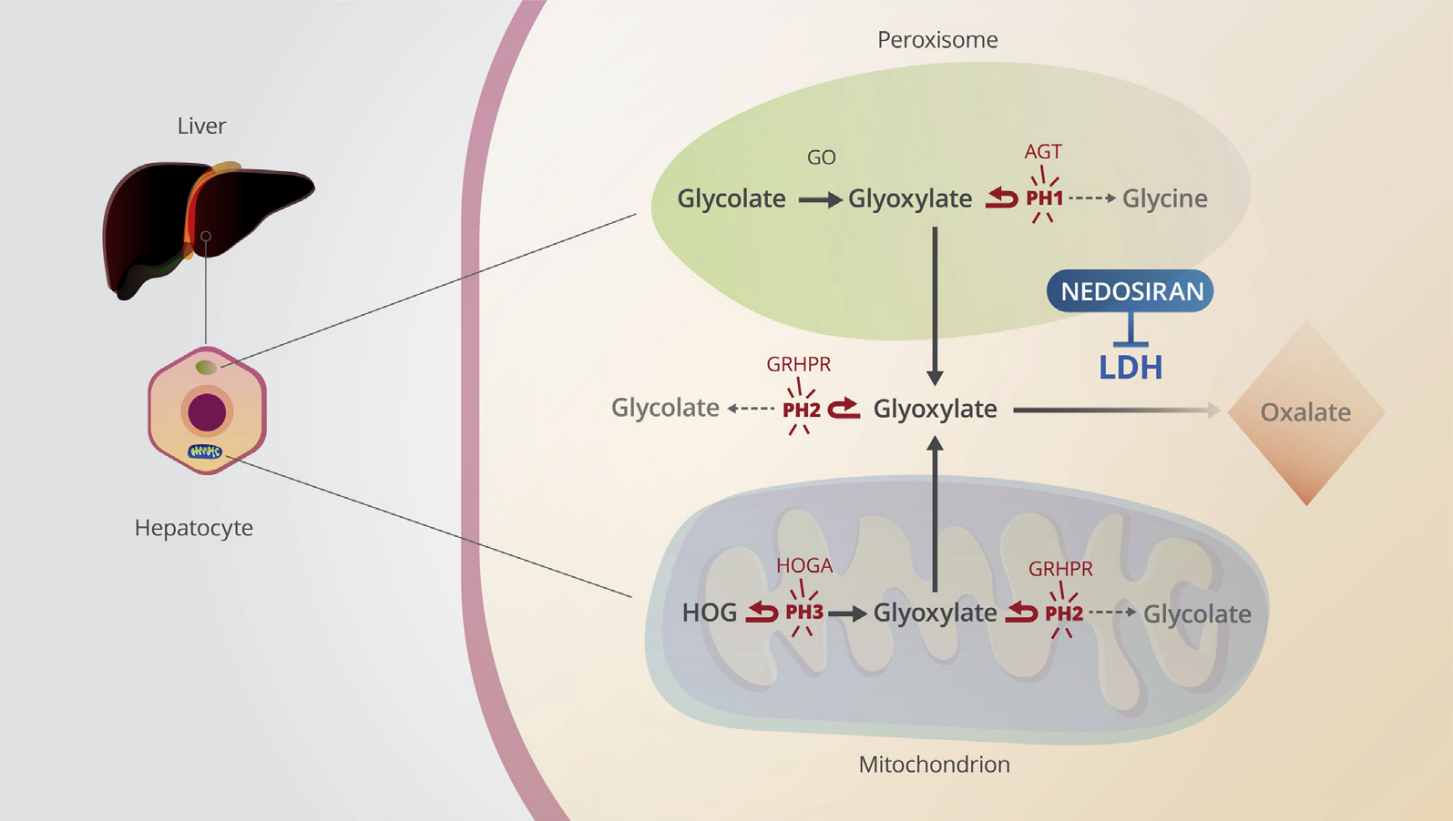
Glyoxylate metabolism in primary hyperoxaluria. Lactate dehydrogenase (LDH)–mediated conversion of glyoxylate to oxalate in the liver is the ultimate step resulting in oxalate overproduction in primary hyperoxaluria types 1, 2, and 3. Nedosiran is an investigational RNA interference therapy designed to inhibit hepatic LDH (encoded by *LDHA*). AGT, alanine:glyoxylate aminotransferase; GO, glycolate oxidase; GRHPR, glyoxylate reductase/hydroxypyruvate reductase; HOG, 4-hydroxy-2-oxoglutarate; HOGA, 4-hydroxy-2-oxoglutarate aldolase.

Common to all PH types is the accumulation of glyoxylate, which is metabolized to oxalate in the liver. Glyoxylate is normally converted to either glycine (by alanine:glyoxylate aminotransferase), glycolate (by glyoxylate reductase/hydroxypyruvate reductase), or oxalate (by lactate dehydrogenase [LDH]) in the liver. In PH1 and PH2, reduced alanine:glyoxylate aminotransferase or glyoxylate reductase/hydroxypyruvate reductase activity leads to an increased buildup of glyoxylate and a resultant overproduction of oxalate by LDH. In PH3, 2 mechanisms for oxalate overproduction have been proposed, both of which ultimately lead to the accumulation of cytosolic glyoxylate and its subsequent conversion to oxalate by LDH.^2^^,^^6^ Oxalate cannot be metabolized further and is almost exclusively excreted by the kidneys, where it exerts its hazardous effects because of precipitation of its calcium salt (calcium oxalate or CaOx).^2^^,^^7^

The earliest and most common clinical manifestations of all PH types are nephrolithiasis and progressive nephrocalcinosis resulting from the deposition of highly insoluble CaOx crystals throughout the kidney.^2^^,^^6^ This may lead to progressive renal damage and chronic kidney disease, which often culminates in renal failure.^2^^,^^8^ PH1 is the most severe of all PH types. It is estimated that 20% to 50% of patients with PH (mainly in PH1) have advanced renal disease, and 27% have renal failure at the time of or before PH diagnosis.^6^^,^^9^ As kidney injury progresses, a second phase of insult occurs when the kidneys are unable to excrete the load of oxalate because of the declining filtration rate. This causes oxalate buildup in the plasma and deposition of CaOx crystals in virtually all tissues in a process called systemic oxalosis, which can be life-threatening.^2^^,^^6^ Most current PH therapies are conservative measures and are poorly effective. A liver-kidney transplant is currently the only option to prevent systemic oxalosis in patients progressing to renal failure in PH1.^10^ In PH2, isolated kidney transplantation is the method of choice, although a combined liver-kidney transplant may be required in some severe cases.^11^

The lack of approved pharmacologic therapies for PH^1^ poses a major unmet medical need. Therapies targeted at glyoxylate metabolism to reduce hepatic oxalate burden have gained interest in the PH arena. One such target is LDH, a tetrameric enzyme primarily known for its function in the interconversion of pyruvate and lactate via the Cori cycle.^12^, ^13^, ^14^ In PH, LDH plays a critical role in hepatic oxalate production. The 2 most common subunits of LDH are LDHA (or M subunit) and LDHB (or H subunit), which are encoded by the *LDHA* and *LDHB* genes, respectively. These 2 subunits can assemble into 5 different tetrameric combinations or isozymes (LDH1–LDH5). The LDHA subunit is predominantly expressed in the muscle and liver, whereas LDHB is the predominant form in the heart, spleen, kidney, brain, and erythrocytes.^15^ LDH plays a central role in oxalate metabolism in the liver (Figure 1) because it is the key enzyme responsible for the conversion of glyoxylate to oxalate,^16^, ^17^, ^18^, ^19^ thereby controlling the ultimate step of oxalate production and its subsequent urinary excretion. Liver-specific knockdown of *LDHA* and the resultant reduction of LDHA, therefore, offers the potential to reduce hepatic oxalate production and urinary oxalate excretion in all genetically defined types of PH.^20^

Nedosiran (formerly DCR-PHXC) is an investigational ribonucleic acid interference (RNAi) therapy^21^ being developed as a treatment for PH. Nedosiran is a synthetic, double-stranded RNA oligonucleotide (i.e., small interfering RNA [siRNA]) designed to target the mRNA encoding *LDHA.* It is being evaluated in clinical trials for the treatment of all 3 genetically defined types of PH (PH1, PH2, and PH3). The 2-part phase I trial, PHYOX1 (ClinicalTrials.gov identifier NCT03392896) has been completed. The first part of the trial (group A; *N* = 25) was a placebo-controlled, single-blind trial in healthy volunteers (HVs), while the second part (group B; *N* = 18) was an open-label trial in patients with genetically confirmed PH (PH1 and PH2).

Liver-specific targeting of nedosiran is achieved by conjugating the RNA oligonucleotide to *N-*acetyl galactosamine (GalNAc) aminosugar residues. This GalNAc conjugation enables specific binding to the asialoglycoprotein receptors (ASGPRs), which are predominantly expressed on the surface of hepatocytes.^22^, ^23^, ^24^, ^25^ Once preferentially taken up by the liver, nedosiran engages the natural endogenous RNAi machinery to selectively reduce *LDHA* mRNA levels, thereby reducing the production of LDHA protein and LDH enzyme in hepatocytes. Ultimately, the final step responsible for the overproduction of hepatic oxalate is inhibited. The proof-of-concept that *LDHA*-targeted siRNAs can reduce oxalate production has been demonstrated using animal models of PH1 and PH2.^20^^,^^26^

While selective inhibition of hepatic LDH enzyme promises to be an elegant therapeutic target for all genetically defined types of PH, it is prudent to prove that inhibition of the hepatic isozyme does not produce unintended consequences in off-target tissues, such as muscles. Nonclinical testing of siRNAs that target hepatic *LDHA*^20^^,^^26^ has provided evidence to support the hypothesis that liver-specific *LDHA* inhibition using GalNAc-conjugated siRNAs, such as nedosiran, produces no apparent adverse effects in off-target (nonhepatic) tissues. Herein, we provide clinical data to corroborate the safety of nedosiran-mediated hepatic *LDHA* inhibition by 1) defining the human phenotype of congenital LDHA deficiency (LDHAD) based on a comprehensive review of cases published in the literature and 2) correlating the LDHAD phenotype with clinical data in the form of biomarker levels and muscle-related adverse events (AEs) in HVs (group A) randomized to receive single-dose nedosiran or placebo in the phase I trial. People with congenital LDHAD lack *LDHA* expression in all tissues. They therefore represent the most extreme scenario, which we can use as a baseline to assess the effect of hepatic *LDHA* inhibition.

## Methods

### Literature Review

A comprehensive review of literature was performed on PubMed with no time limits on publication date using the following search terms: L-lactate dehydrogenase/deficiency, L-lactate dehydrogenase contains: dehydrogenase, L-lactate, L lactate dehydrogenase, lactate dehydrogenase, dehydrogenase, lactate. English language articles that reported human LDHAD cases were included. Foreign language articles were included if their abstracts were in English and provided relevant patient information on LDHAD. The following types of articles were excluded from further review: articles describing other subtype deficiencies (e.g., LDHB/H) and articles reporting nonclinical data.

### PHYOX1 Study Design

PHYOX1 (EudraCT No: 2017-003534-89; Clinicaltrials.gov identifier NCT03392896) is a 2-part, phase I, single-ascending dose study of subcutaneous nedosiran in HVs (group A) and adult patients with PH1 or PH2 (group B) conducted between November 2017 and November 2019. The primary objective of the group A portion of the study was to evaluate the safety and tolerability of nedosiran. Clinical laboratory data and musculoskeletal AE data from group A will be discussed in this manuscript, and methods applying only to that portion of the trial will be presented here. The remainder of the PHYOX1 clinical trial will be described in a separate article.

Group A was designed as a placebo-controlled, participant-blind, single ascending-dose study wherein participants were randomized into 5 sequential dose cohorts (0.3, 1.5, 3.0, 6.0, and 12.0 mg/kg nedosiran or placebo) with 5 participants (3 active, 2 placebo) in each cohort (Supplementary Figure S1). Male or female HVs between 18 and 55 years of age (inclusive) with a body mass index of 19.0 to 32.0 kg/m^2^ (inclusive) were eligible to take part in the study if they met all eligibility criteria set forth in the study protocol (Supplementary Table S1). Participants were enrolled on day 0 and randomized on day 1 to receive a single dose of nedosiran or placebo. Participants were discharged from the study site on day 3 and returned to the site at specified time points through day 29 (end of study). The study protocol and amendments were approved by an independent ethics committee, and the study was conducted according to the International Conference on Harmonization guidelines and relevant country-specific laws and regulations.

### PHYOX1 Safety Analyses

Safety analyses were performed at scheduled time points throughout the study and included physical examination, vital signs, electrocardiograms, standard clinical laboratory testing, concomitant medications, and AEs (coded using MedDRA version 21.0). Of these, AEs classified under the “musculoskeletal and connective tissue disorders” system organ class (SOC) were discussed herein. Blood samples were collected from participants on days 0 (baseline), 2, 8, 15, 22, and 29 (end of study) for lactate, pyruvate, and creatine kinase (CK) analyses. Fasted samples were obtained at the clinical site at approximately the same time of the day on days 0, 2, 15, and 29. Samples on days 8 and 22 were outpatient samples, so fasting was variable and not always as long as for samples obtained at the clinical site. For lactate analysis, blood was collected into a 1.2-mL fluoride/ethylenediamine tetraacetic acid monovette and sent for analysis within 15 minutes of sample collection. For pyruvate, blood was collected in a 1.2-mL lithium heparin monovette and transported on wet ice for immediate analysis. For CK, blood was collected in a 4.5-mL plain monovette. Serum was separated by centrifuging at 4 °C for 10 minutes using centrifugal force equal to 2000 *g*, transferred into a 2.5-mL false bottom propylene tube, and stored refrigerated at approximately 4 °C pending analysis. Lactate, pyruvate, and CK levels in plasma/serum samples were assessed by enzymatic assays using cobas c analyzers (Roche Diagnostics, Indianapolis, Indiana, USA) according to the manufacturer’s instructions and methods reported in the literature.^27^

### Statistical Analyses

Graphs were prepared using Prism software (version 8.4.3; GraphPad Software, San Diego, CA). All statistical analyses were performed using SAS software (version 9.4; IBM Corp., Chicago, IL). The Wilcoxon rank sum test was used to compare biomarker data between nedosiran and placebo groups. Biomarker changes from days 0 to 29 within each group were compared using the Wilcoxon signed rank test. The criterion for statistical significance was *P* < 0.05.

## Results

### Literature Review: Patients With Congenital LDHAD Do not Show Signs of Liver Manifestations

The literature search yielded 137 articles (see Methods for search criteria), of which 20 articles were relevant to human LDHAD cases.^28^, ^29^, ^30^, ^31^, ^32^, ^33^, ^34^, ^35^, ^36^, ^37^, ^38^, ^39^, ^40^, ^41^, ^42^, ^43^, ^44^, ^45^, ^46^, ^47^ Multiple articles reported on the same patient(s) with LDHAD. To reduce duplication of patient numbers, unique cases were identified and matched based on cross-referencing of citations and family pedigree charts (to the extent possible in a literature review setting). Based on this process, 14 unique patients (6 female and 8 male) with congenital LDHAD were identified: 11 cases in 7 families in Japan, 1 case in an Italian family, and 2 cases in 2 U.S. families.

**Figure 2:**
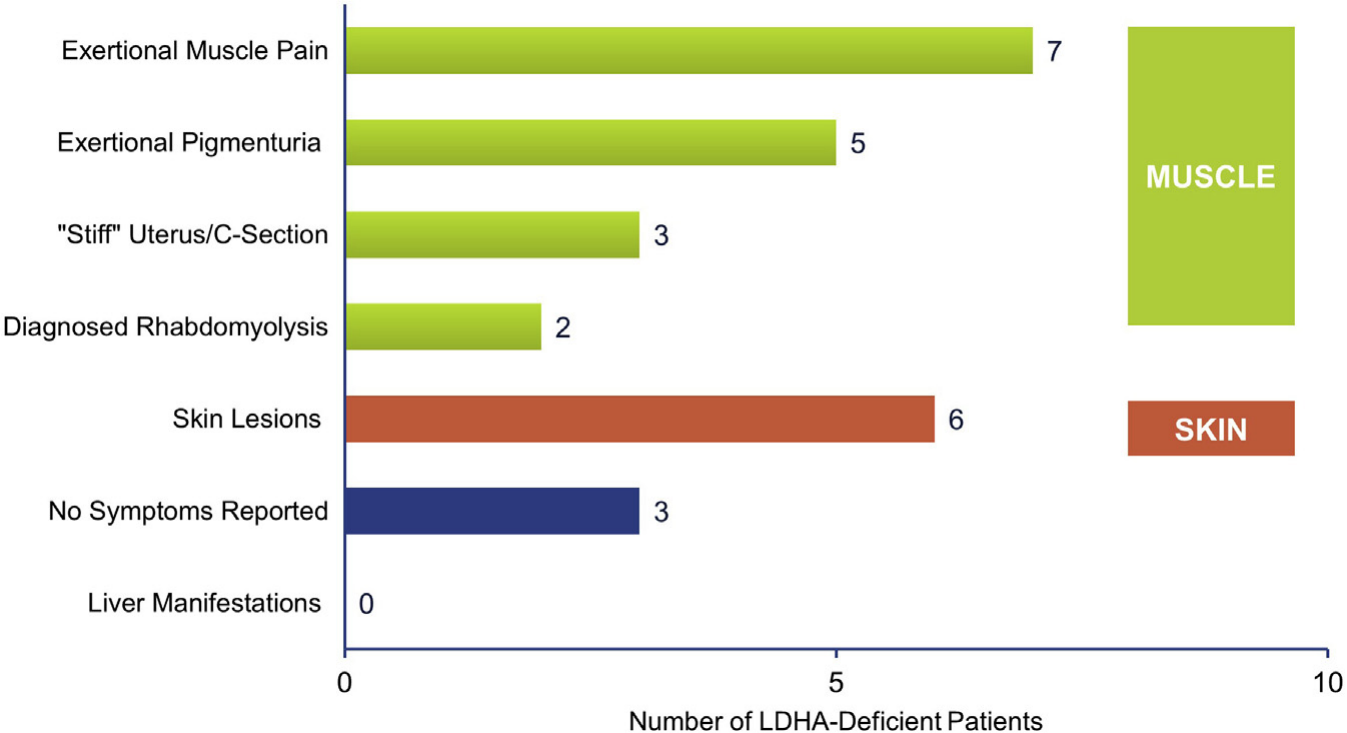
Patients with lactate dehydrogenase A (LDHA) deficiency: symptoms reported in the published literature. The symptoms of LDHA deficiency reported for the 14 patients were primarily muscle-related (green bars). Multiple symptoms were reported in a single patient in some cases.

Figure 2 categorizes the symptoms reported in patients with LDHAD identified in the literature. Of the 23 symptoms reported, 74% (17/23) were related to some form of muscular manifestation and the remaining 26% (6/23) were skin-related. There were no liver manifestations reported among any patients. Exertional muscle pain was the most common symptom followed by skin lesions and exertional pigmenturia (myoglobinuria and hemoglobinuria). Three of the 6 female patients (50%) reported uterine stiffness during pregnancy. Chest pain was reported in 1 patient but not directly linked to LDHAD by the authors.^42^ In 3 patients, no symptoms were reported in these article(s).

### Literature Review: Alterations in Lactate, Pyruvate, and CK Levels in Patients With LDHAD

Symptoms were typically more common in patients with LDHAD when they were engaged in various types of exercise (e.g., judo wrestling, pole climbing, 100-meter dash). In general, these patients did not report symptoms under nonexercise conditions (Table 1).^30^^,^^31^ At rest, plasma levels of both lactate and pyruvate in patients with LDHAD were found to be similar to those in non-LDHAD control subjects. When subjected to ischemic exercise testing of the forearm, plasma pyruvate was increased significantly in these patients compared with control subjects. The expected increase in lactate levels was reduced compared with control subjects.^28^^,^^30^^,^^31^^,^^33^ As further evidence of predominant skeletal muscle involvement, a marked increase in plasma CK relative to control subjects was also observed when challenged under ischemic exercise conditions.^28^

**Table 1:** Summary of lactate dehydrogenase A deficiency phenotype defined by the literature

Key takeaways from the literature review of lactate dehydrogenase A deficiency
• No liver manifestations
• Patients largely asymptomatic except under exercise conditions
• Normal lactate levels at rest (range 0.4**–**0.6 mM)^a^
• Normal pyruvate levels at rest (range 0.07**–**0.09 mM)^a^^,^^b^
• Elevations in creatine kinase levels at rest noted in some cases (range 48**–**26,290 U/L)^c^
• Marked elevation of plasma pyruvate (range 0.68**–**0.88 mM)^d^ and creatine kinase under exercise challenge
• Blunted elevation of plasma lactate (range 3.4**–**4.0 mM)^d^ under exercise challenge

a Based on a single article^33^ reporting numerical values of plasma lactate and pyruvate on 4 patients.

b Significant pyruvate elevations were noted in a single patient during her third trimester of pregnancy; her lactate levels were “almost within normal range” as described by the authors.^39^

c Elevation in creatine kinase levels at rest were noted in 3 articles.^30^^,^^32^^,^^43^

d Based on a single article^33^ reporting numerical values of plasma lactate and pyruvate on 4 patients subjected to incremental exercise on a bicycle ergometer; creatine kinase values were not reported.

### PHYOX1: HVs Treated With Single-Dose Nedosiran Showed no Alterations in Plasma Lactate, Pyruvate, or CK Levels

The first part (group A) of the PHYOX1 trial was a placebo-controlled, participant-blind, single ascending dose study. Group A enrolled 25 HVs at a single site, all of whom completed the study. Five cohorts were dosed at 0.3, 1.5, 3.0, 6.0, or 12.0 mg/kg of nedosiran or placebo (3:2 randomization; Table 2). The mean age of all participants was 33.2 years (range, 19–55 years). The mean body mass index of all participants was 25.4 kg/m^2^.

**Table 2:** Demographic characteristics of PHYOX1 group A (healthy volunteers)

Characteristic	Placebo (*N* = 10)	Nedosiran, mg/kg						All participants (*N* = 25)
		0.3 (*n* = 3)	1.5 (*n* = 3)	3.0 (*n* = 3)	6.0 (*n* = 3)	12.0 (*n* = 3)	Overall (*n* = 15)	
Age (y)								
Mean (SD)	35.5 (9.9)	28.7 (5.7)	28.0 (7.2)	37.3 (17.5)	35.3 (10.5)	29.3 (6.4)	31.7 (9.6)	33.2 (9.7)
Sex, n (%)								
Male	8 (80.0)	2 (66.7)	1 (33.3)	1 (33.3)	2 (66.7)	0	6 (40.0)	14 (56.0)
Female	2 (20.0)	1 (33.3)	2 (66.7)	2 (66.7)	1 (33.3)	3 (100)	9 (60.0)	11 (44.0)
BMI (kg/m^2^)								
Mean (SD)	26.47 (3.28)	26.37 (4.27)	24.36 (3.31)	26.93 (3.14)	21.96 (1.41)	23.81 (2.53)	24.69 (3.20)	25.40 (3.29)

BMI, body mass index; SD, standard deviation.

**Figure 3:**
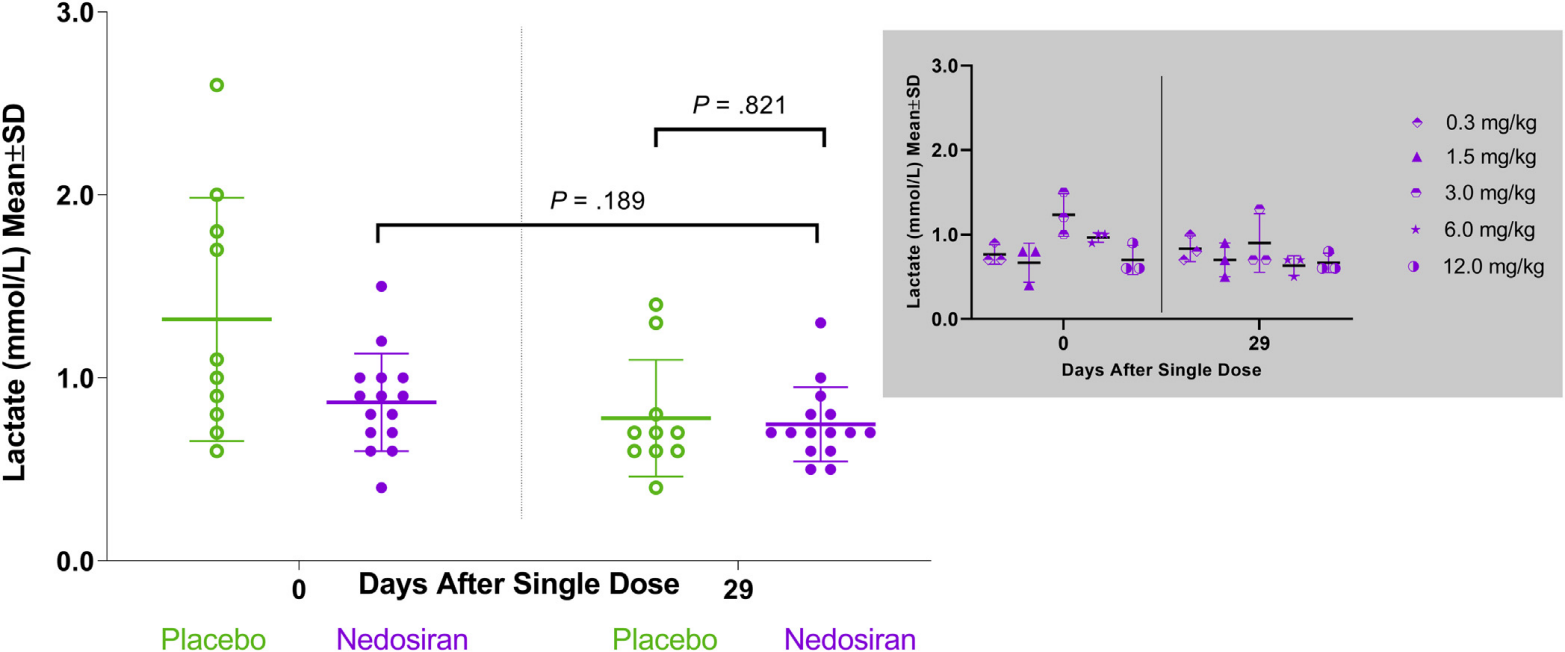
Plasma lactate distribution in healthy volunteers. Scatter plot showing plasma lactate levels in healthy volunteers at baseline (day 0) and at the end of the study (day 29) in placebo and nedosiran groups. Inset graph (in gray) shows plasma lactate distribution by nedosiran dose. SD, standard deviation

**Figure 4:**
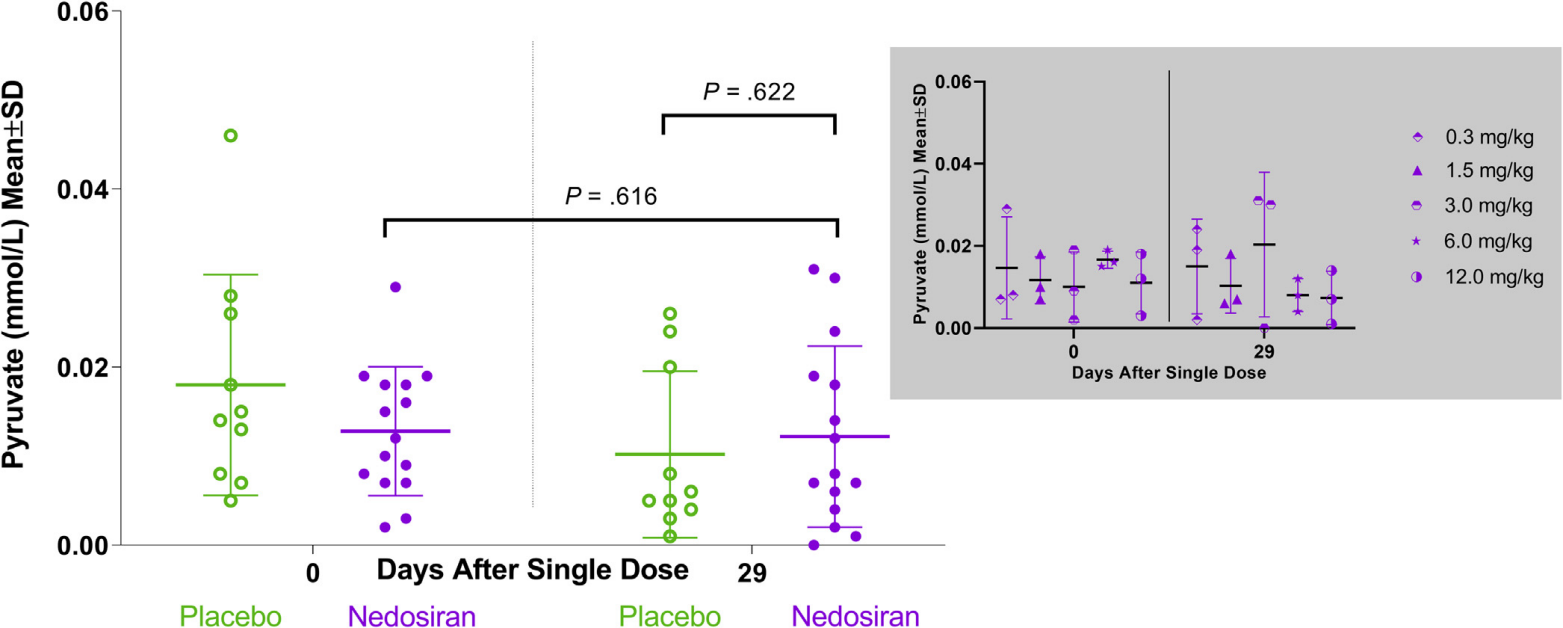
Plasma pyruvate distribution in healthy volunteers. Scatter plot showing plasma pyruvate levels in healthy volunteers at baseline (day 0) and at the end of the study (day 29) in placebo and nedosiran groups. Inset graph (in gray) shows plasma pyruvate distribution by nedosiran dose. SD, standard deviation.

**Figure 5:**
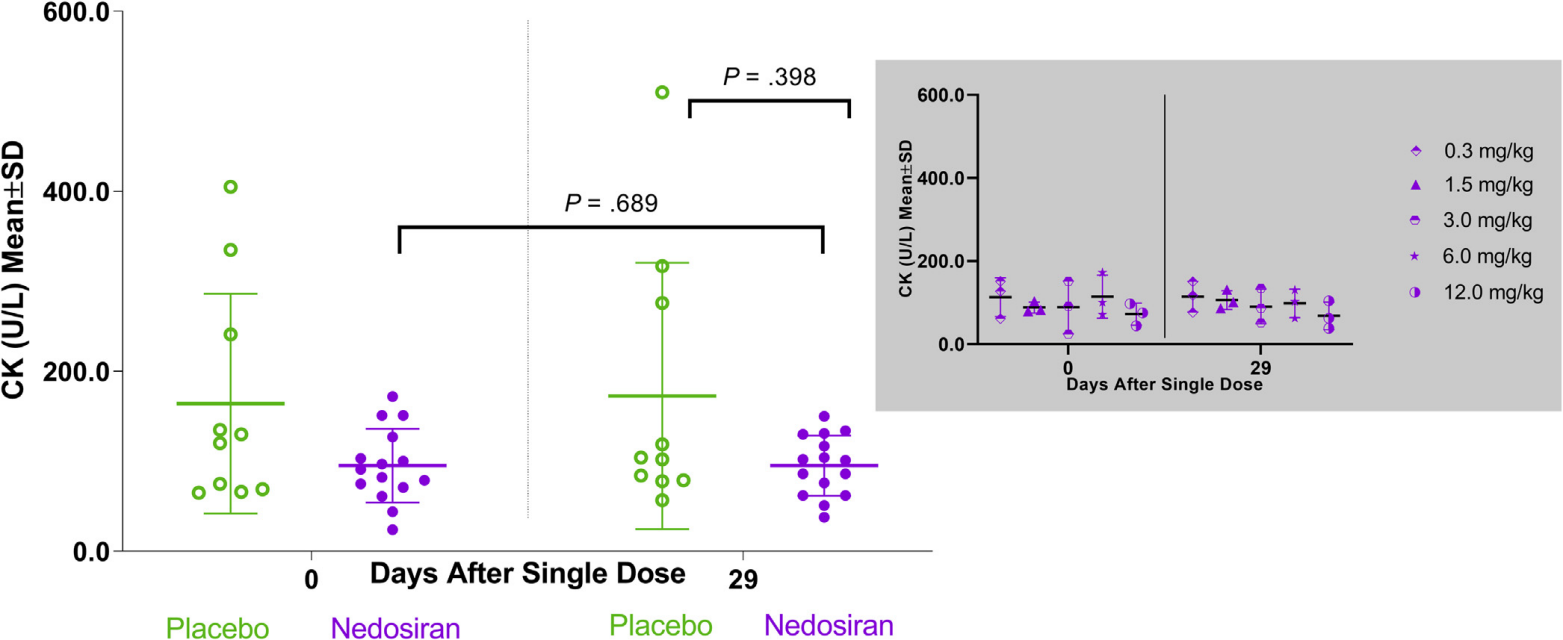
Plasma creatine kinase (CK) distribution in healthy volunteers. Scatter plot showing plasma CK levels in healthy volunteers at baseline (day 0) and at the end of the study (day 29) in placebo and nedosiran groups. Inset graph (in gray) shows plasma CK distribution by nedosiran dose. SD, standard deviation.

**Figure 6:**
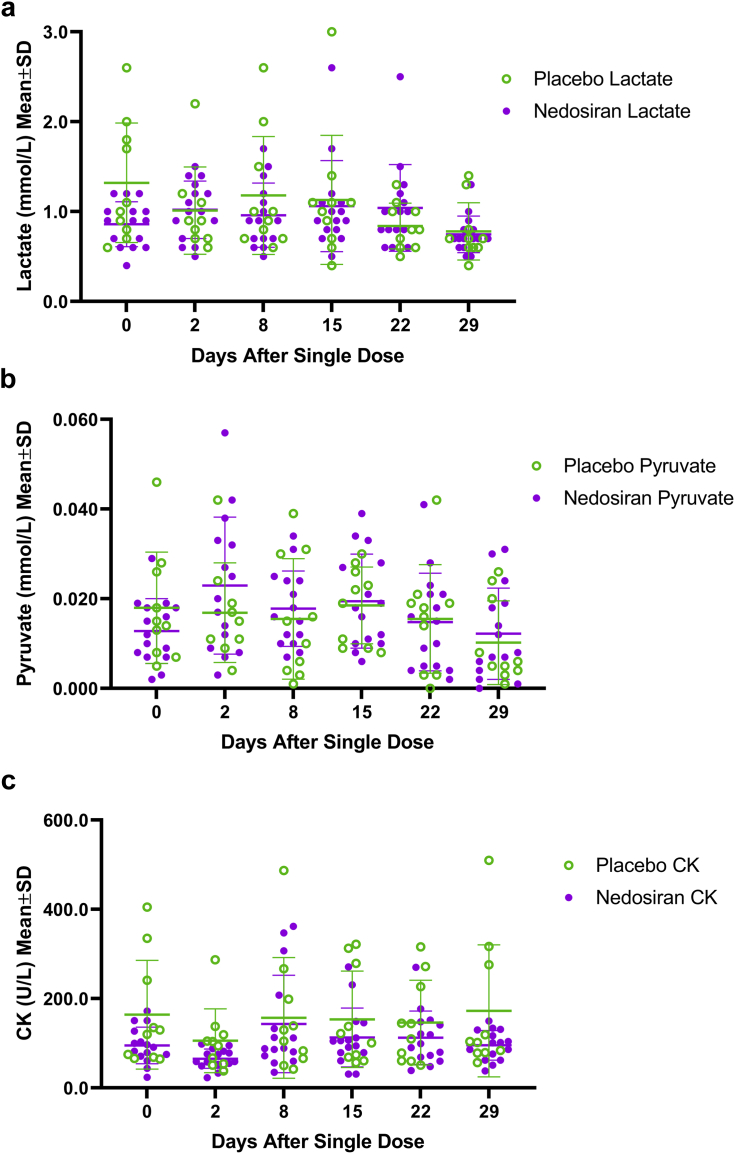
Superimposed lactate, pyruvate, and creatine kinase (CK) distribution profiles in healthy volunteers. Superimposed scatter plots showing (a) lactate, (b) pyruvate, and (c) CK profiles in the nedosiran (*N* = 15) and placebo (*N* = 10) groups at timepoints between days 0 and 29 (days 2, 8, 15, and 22). One participant had a missing lactate value, and another participant had a missing pyruvate value, both on day 2 within the placebo group. SD, standard deviation.

Among HVs treated with a single dose of nedosiran (0.3–12.0 mg/kg), there were no significant changes in plasma lactate, pyruvate, or CK levels compared with placebo at the end of study (day 29; Table 3). Within the placebo group, changes from day 0 (baseline) to day 29 were significant for lactate and pyruvate, but not for CK. No significant changes were observed from day 0 to day 29 for any of the 3 biomarkers within the nedosiran group (Figure 3, Figure 4, Figure 5). There were also no apparent biomarker trends based on nedosiran dose levels (Figure 3, Figure 4, Figure 5, insets). A low sample size (*n* = 3 per group) precludes meaningful statistical analysis at the dose level. Both the nedosiran and placebo groups showed similar lactate, pyruvate, and CK profiles during the course of the study as seen from measurements at timepoints between days 0 and 29 (Figure 6a–c). Mean biomarker values in either group did not exceed the upper limit of normal during the study timeframe (upper limits of normal: lactate, 2.2 mmol/L; pyruvate, 0.13 mmol/L; CK, 469.5 U/L).

**Table 3:** Biomarker values in healthy volunteers

Biomarker and timeline	Placebo (*N* = 10)		Nedosiran (*N* = 15)		*P* value
	Mean (SD)	Median (min, max)	Mean (SD)	Median (min, max)	
Plasma lactate (mmol/L)					
Baseline (day 0)	1.32 (0.66)	1.05 (0.60, 2.60)	0.87 (0.27)	0.90 (0.40, 1.50)	.821^a^
End of study (day 29)	0.78 (0.32)	0.70 (0.40, 1.40)	0.75 (0.20)	0.70 (0.50, 1.30)	.189^b^ .027^c^
Plasma pyruvate (mmol/L)					
Baseline (day 0)	0.018 (0.012)	0.014 (0.005, 0.046)	0.013 (0.007)	0.012 (0.000, 0.029)	.622^a^
End of study (day 29)	0.010 (0.009)	0.006 (0.001, 0.026)	0.012 (0.010)	0.008 (0.000, 0.031)	.616^b^ .023^c^
Plasma creatine kinase (U/L)					
Baseline (day 0)	164.10 (121.81)	125.00 (65.00, 405.00)	95.20 (41.00)	91.00 (24.00, 172.00)	.398^a^
End of study (day 29)	172.60 (147.90)	103.00 (57.00, 510.00)	95.33 (33.38)	101.00 (38.00, 150.00)	.689^b^ 1.000^c^

SD, standard deviation.

a Day 29 nedosiran versus day 29 placebo.

b Day 0 nedosiran versus day 29 nedosiran.

c Day 0 placebo versus day 29 placebo.

### PHYOX1: No Drug-Related Musculoskeletal AEs Were Observed in the Single-Dose Nedosiran Group

Seven participants in group A (3 in placebo; 4 in nedosiran) experienced ≥1 AE, all of which were of mild or moderate severity. We were interested in assessing the effect of nedosiran on off-target tissues, specifically the muscles. Therefore, AEs classified under musculoskeletal and connective tissue disorders SOC were considered relevant (other AEs will be discussed in a separate article on the entire PHYOX1 trial). Musculoskeletal AEs were experienced by 2 participants in the nedosiran group (Table 4), both of which (back pain and myalgia) were considered by the investigator to be unrelated to the study drug. Both events resolved within 3 days of onset and appeared to have no clinical sequelae.

**Table 4:** Musculoskeletal adverse events in healthy volunteers

System organ class and preferred term	Placebo (*N* = 10)	Nedosiran, mg/kg					
		0.3 (*n* = 3)	1. 5 (*n* = 3)	3.0 (*n* = 3)	6.0 (*n* = 3)	12.0 (*n* = 3)	Overall (*n* = 15)
Participants with ≥1 TEAE, n (%), no. of TEAEs	3 (30.0), 4	0	0	1 (33.3), 1	2 (66.7), 3	1 (33.3), 2	4 (26.7), 6
Musculoskeletal and connective tissue disorders	0	0	0	0	1 (33.3), 1	1 (33.3), 1	2 (13.3), 2
Back pain	0	0	0	0	1 (33.3), 1	0	1 (6.7), 1
Myalgia	0	0	0	0	0	1 (33.3), 1	1 (6.7), 1

TEAE, treatment-related adverse event.

Adverse events were coded using MedDRA version 21.0. TEAEs are events that occurred or worsened on or after the first dose of study drug. Participants are counted once for each system organ class and once for each preferred term.

## Discussion

PH is a family of rare, autosomal recessive genetic disorders of glyoxylate metabolism that lead to hepatic overproduction of oxalate and progressive renal damage, which may culminate in renal failure and systemic oxalosis.^2^^,^^6^ LDH is the final common enzyme responsible for the conversion of glyoxylate to oxalate in all genetically defined types of PH, which makes it an attractive therapeutic target for fulfilling an unmet need in all known types of PH.^20^

Nedosiran is an investigational RNAi therapy (administered monthly via subcutaneous injections) designed to inhibit hepatic *LDHA.* It is intended to reduce the expression of *LDHA* and the resultant LDH activity in the liver by harnessing the natural RNAi pathway. GalNAc conjugation achieves liver-specific targeting of nedosiran by binding to ASGPRs. Although ASGPRs have been detected in nonhepatic tissues, their expression on hepatocytes far exceeds that in other locations, and ASGPRs have no known role elsewhere in the body.^22^, ^23^, ^24^, ^25^ Therefore, in theory and by design, it would be unlikely to see extrahepatic delivery of these siRNAs. Nevertheless, it is imperative to demonstrate the lack of RNAi effects in off-target tissues, specifically the muscle (where *LDHA* is also expressed, along with the liver). Animal studies by Lai *et al.*^20^ and Wood *et al.*^26^ have demonstrated the lack of such off-target effects; both studies found that GalNAc-conjugated siRNAs achieved liver-specific knockdown of *LDHA* in mice with no significant alterations to its expression in other tissues, such as the muscle, skin, kidneys, heart, or uterus. Lai *et al.*^20^ also noted liver-specific knockdown of *LDHA* in nonhuman primates and chimeric mice with humanized livers, which provides an additional level of evidence for liver-specific RNAi delivery across species.

To further explore the lack of off-target effects with siRNAs, we reviewed the data from animal studies against the human phenotype of congenital LDHAD, which represents the most extreme scenario of loss of *LDHA* expression. The human LDHAD phenotype was defined based on a comprehensive review of medical literature spanning >35 years, which identified a total of 14 such patients worldwide. This deficiency presents with a predominantly muscle-related phenotype with some reports of skin lesions. Interestingly, none of the patients with LDHAD had liver manifestations. We therefore deduced that evidence of any off-target effects would manifest in the skeletal muscles. Lai *et al.*^20^ ruled out such manifestations as they observed normal muscle performance when *Ldha-*knockdown mice were subjected to exercise in a treadmill endurance test. No signs of exercise-induced myopathy were noted, unlike the human counterparts with LDHAD. Emerging data from repeat dose toxicity studies in nonhuman primates (monthly dosing of *LDHA* siRNA for ≤9 months) also show no adverse skeletal muscle effects (unpublished data). In keeping with the lack of liver manifestations noted in the human phenotype, animal data also show no liver histopathology or acute liver toxicity with siRNA-*LDHA* inhibition. No changes in body composition or weight loss were noted, reinforcing the overall safety of this approach.^20^^,^^26^

Another aspect of *LDHA* inhibition is its potential effect on the Cori cycle and related metabolic pathways, such as gluconeogenesis. Patients with LDHAD showed a marked elevation of plasma pyruvate and CK because of impaired glycolysis under exercise challenge conditions. Wood *et al.*^26^ noted a significant rise in both liver and plasma pyruvate levels under nonexercise conditions in a PH1 mouse model treated with liver-specific siRNA targeted at *LDHA*. In contrast to these findings, Lai *et al.*^20^ noted that liver-specific siRNA-*LDHA* inhibition in mice did not result in significant elevations of plasma pyruvate under nonexercise conditions and during exercise. There were no signs of lactic acidosis, as suggested by the absence of plasma lactate elevations in mice. Similarly, liver-specific *LDHA* inhibition did not result in an elevation of lactate in nonhuman primates. In addition, mice showed normal muscle function during exercise, implying an uninterrupted glucose resupply from liver to muscles, indicating a properly functioning hepatic gluconeogenesis pathway.^20^ The lack of significant increases in plasma lactate and pyruvate after hepatic *LDHA* inhibition was also corroborated by clinical data obtained from group A of the PHYOX1 trial. HVs treated with up to 12 mg/kg of nedosiran showed no significant changes in plasma lactate, pyruvate, or CK levels compared with placebo during the clinical trial observation period. There were no significant changes in lactate, pyruvate, or CK within the nedosiran group between baseline and end of study (day 29), further confirming the lack of any systemic effects of hepatic *LDHA* inhibition in humans (especially its role in the Cori cycle and other interconnected metabolic pathways). The statistically significant changes in lactate and pyruvate within the placebo group could be attributed to the variability in the baseline values within the group. These changes are not thought to be clinically meaningful, which is corroborated by the fact that the participants in the placebo group were asymptomatic and did not experience any adverse events that would be attributable to such a reduction in either pyruvate or lactate. Overall, an analysis of the existing nonclinical data and the current phase I clinical trial data show that RNAi-mediated *LDHA* inhibition in the liver appears to be safe for disease targeting. Presumably, this inhibition has a predominant impact on oxalate formation as opposed to the interconversion of lactate and pyruvate. None of the HVs experienced any drug-related musculoskeletal AEs after treatment with nedosiran, which also supports the specificity of RNAi action and the lack of unintended consequences in off-target tissues.

We acknowledge that the biochemical findings and lack of drug-related musculoskeletal AEs presented here are based on a single dose of nedosiran in HVs alone. The AE data on patients with PH are beyond the scope of this article and will be discussed in a follow-on article reporting the totality of results from the PHYOX1 study. The biomarker data presented here were collected under clinical trial conditions that were not intended to replicate exercise challenge conditions. The transient nature of the pyruvate and lactate alterations after exercise made it impractical to capture in a clinical setting. Animal models are an alternative, on which we based our conclusions for exercise-related biochemical effects of *LDHA* inhibition. It is also important to note that participants were able to perform activities of daily living, without any drug-related AEs after the administration of nedosiran. The ongoing pivotal (PHYOX2)^48^ and open-label extension (PHYOX3)^49^ trials are multidose trials evaluating nedosiran. They are designed to capture safety, including muscle-related AEs and CK elevations, and efficacy assessments following chronic nedosiran dosing. Thus, they are prospectively monitoring the chronic effects of *LDHA* inhibition.

In summary, an analysis of the published nonclinical and recent clinical evidence supports RNAi-mediated *LDHA* inhibition as a potentially safe therapeutic mechanism for all genetic variants of PH.

## Disclosure

CBL is a consultant for Dicerna Pharmaceuticals, Inc. (Lexington, MA) and Allena Pharmaceuticals, Inc. (Newton, MA). GA reports personal fees and nonfinancial support from Alexion Pharmaceuticals, personal fees and nonfinancial support from Recordati Rare Diseases, Advicenne, nonfinancial support from Kyowa Kirim, personal fees from Dicerna Pharmaceuticals, personal fees and other from Chiesi, outside the submitted work. KB, BDB, BH, and RR are employees of Dicerna Pharmaceuticals, Inc. BDB reports patents issued/pending relevant to the submitted work.

## Supplementary Material

Supplementary Material (PDF)

**Figure S1.** PHYOX1-group A study design schematic.

**Table S1.** Inclusion and exclusion criteria for PHYOX1-group A participants.

**CONSORT statement.**

## Footnotes

1 Lumasiran, an RNAi therapy targeting hepatic GO, was recently approved as a treatment for PH1.
